# A case report of end-stage achalasia: Conservative option as the new surgical standard

**DOI:** 10.1016/j.ijscr.2023.108762

**Published:** 2023-08-30

**Authors:** Mohamed Mehdi Trabelsi, Neirouz Kammoun, Souhir Nasseh, Mohamed Ali Chaouch, Hichem Jerraya

**Affiliations:** a Department B of Surgery, Charles Nicolle Hospital, Tunis, Tunisia; b General Surgery Department, Fattouma Bourguiba Hospital, Monastir, Tunisia

**Keywords:** End-stage achalasia, Laparoscopic Heller-Dor, Esophagectomy, Case report

## Abstract

**Introduction and importance:**

End-stage achalasia is a rare disease, consisting of a functional impairment of the esophagus which becomes dilated with a sigmoid shape. While esophagectomy was considered for a long time as the principal surgical procedure in end-stage achalasia, recent literature results demonstrate that laparoscopic Heller Dor (LHD) could be an advocated alternative with acceptable functional results.

**Case presentation:**

We present the case of an eighty-three-year-old male, an elderly patient, who had been complaining for one year of dysphagia and general status loss. Endoscopy, manometry then a barium X-ray confirmed end-stage achalasia. The patient had LHD with an improvement of symptomatology post-operatively.

**Clinical discussion:**

Achalasia is a rare disease affecting oesophagal motility. The diagnosis is suggested clinically and confirmed by a wide range of tests notably esophagogastroduodenoscopy, barium swallow and manometry. The diagnosis of achalasia is classically made by demonstrating impaired relaxation of the lower oesophagal sphincter and absent peristalsis in the oesophagal manometry. Esophagogastroduodenoscopy is made mainly to eliminate the diagnosis of oesophagal cancer. Barium swallow, however, is done to appreciate the impact of achalasia on the rest of the esophagus.

**Conclusion:**

Our case highlights the satisfying results after an LHD which is an alternative to esophagectomy especially in elderlies with high risk.

## Highlights

• While esophagectomy was considered for a long time as the principal surgical procedure in end-stage achalasia
• Literature demonstrate that laparoscopic Heller Dor could be an advocated with acceptable functional results.
• Technical detail should be added with a pull-down of the oesophagus to ensure a vertical axis of a sigmoid shape oesophagus.

## 1 Introduction

Achalasia is a rare disease with an estimated annual incidence of 1 case per 100,000/year [1]. It is due to esophageal myenteric plexus dysfunction leading to an impairment of the peristaltic with hyper-pressurization of the cardio-esophageal junction provoking various symptoms [1]. In the last stage of the disease, the esophagus becomes dilated with a sigmoid shape leading to severe symptoms. That is what is referred to the end-stage achalasia [2] or radiological stage IV achalasia. The treatment of such pathology is still debated. In fact, till the last decade, esophagectomy was the main surgical procedure performed for such cases. However, Heller myotomy is more and more attempted because of its relative simplicity with less post–operative issues but with controversial results [3,4]. We report a case of an eighty-three-year-old male who had been complaining of end-stage achalasia with general status loss, and who underwent a Heller myotomy with Dor fundoplication. This manuscript is structured following SCARE criteria [5].

## 2 Case presentation

**Fig. 1:**
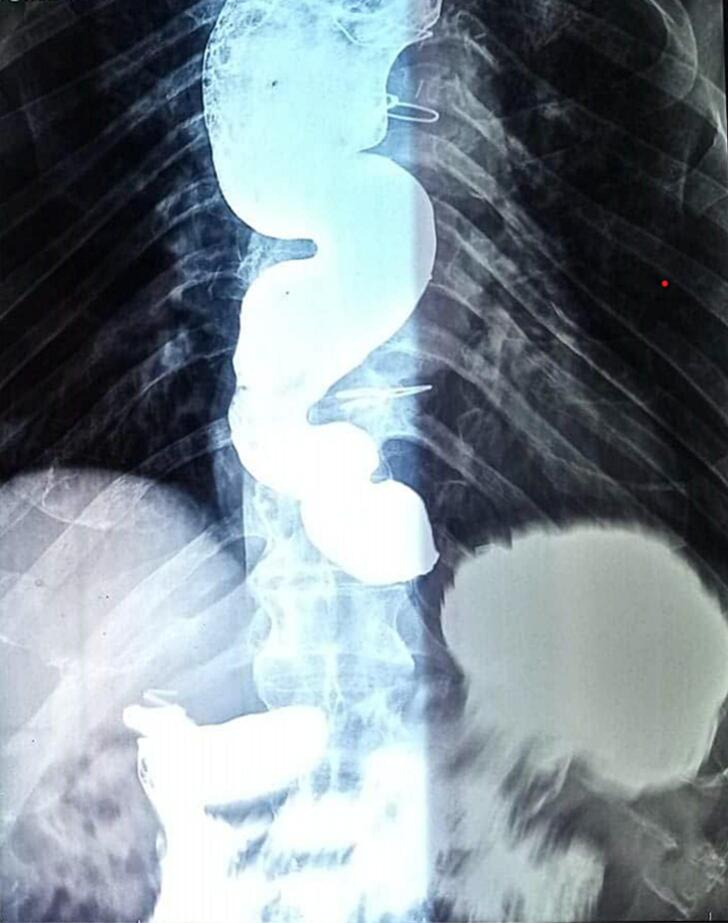
Barium X-ray with a sigmoid shape esophagus.

An eighty-three-year-old male patient with a medical history of a coronary artery bypass had been complaining for one year of dysphagia, regurgitations and general status loss. The patient was only able to swallow small quantities of liquid. The physical examination was not relevant. He was initially explored by an esophagogastroduodenoscopy. It revealed a dilated esophagus with a liquid stasis, cardia incompetence and inflammation of the esophageal mucosa. No tumor, external compression to the esophageal lumen, or even benign polyps existed. A manometry was then performed which revealed an esophageal aperistaltism with high pressure of the lower sphincter at 47 mmHg and an absence of relaxation. The deglutition was not transmitted in 100 % of the attempts. There was also a high gradient pressure between the esophagus and the stomach estimated at 15 mmHg which naturally should be negative. The diagnosis of achalasia stage II, according to the Chicago classification, was made. A barium swallow was performed. It showed a sigmoid sharpening of the esophagus confirming the end-stage achalasia (Fig. 1). The case was discussed in a multi-disciplinary meeting between healthcare staff. Opinions were divided between defenders of esophagectomy and those who were for conservative treatment, which is LHD. In concentration with the patient and considering the medical history, the age of the patient, and the anesthesia risk, we opted for LHD. Before surgery, the patient was put on proton pump inhibitors for esophagitis with good clinical and endoscopic outcomes. The surgical procedure was performed by laparoscopy (Supplementary video): The esophagogastric junction is exposed for 6 to 8 cm proximally, and we did longitudinal myotomy in the anterior esophageal axis using the harmonic scalpel. There was bleeding during the dissection due to esophagitis. The postoperative follow-up was uneventful. After a one-year survey, the patient was seen in the outpatient department. Symptoms were reduced, Eckardt score decreased from 7 to 1, and the patient was satisfied as he could again eat solid food without thoracic pain or regurgitation.

## 3 Discussion

Achalasia is a rare disease affecting esophageal motility [6]. The diagnosis is suggested clinically and confirmed by a wide range of tests notably esophagogastroduodenoscopy, barium swallow and manometry. The diagnosis of achalasia is classically made by demonstrating impaired relaxation of the lower esophageal sphincter and absent peristalsis in the esophageal manometry. Esophagogastroduodenoscopy is made mainly to eliminate the diagnosis of esophageal cancer. Barium swallow, however, is done to appreciate the impact of achalasia on the rest of the esophagus. Classical findings are the distal esophagus tapering in a “bird's beak” configuration with proximal dilation of the organ, sometimes with an air-fluid level, and absence of intra-gastric air. In more advanced cases, severe dilatation with stasis of food and a sigmoid-like appearance can occur [7], and that which defines end-stage achalasia or radiological stage IV achalasia. The natural history of achalasia features a possible continuous evolution of the manometric patterns involved, along with changes in the shape of the esophagus, which becomes gradually enlarged, ultimately acquiring a sigmoid shape [8]. The standard of care for achalasia is laparoscopic Heller cardiomyotomy. This procedure achieves satisfactory and long-standing results in over 85 % of patients [9]. Endoscopic forceful pneumatic dilatation of the cardia has long been the therapy for non-advanced achalasia with good results [5]. In 2010, Inoue et al. [10] described the results of a new endoscopic technique called per-oral endoscopic myotomy (POEM) in 17 patients with esophageal achalasia. They described the endoscopic creation of a submucosal tunnel, which allowed a myotomy by the transection of the circular fibers of the distal esophagus. The most debated indication of POEM and dilation was for young patients. In our case, the patient was 83 years old and a non-surgical POEM myotomy approach was suggested. However, it was not feasible in our institution. The myotomy was carried down to include 2 cm of the gastric wall. However endoscopic treatment hasn't already proven its worth in end-stage achalasia. At this stage of the disease, opinions are almost the same on the importance of surgery but divided between defenders of esophagectomy and those who were for LHD. Valentina et al. [11] highlighted this dilemma and concluded with similar results in terms of dysphagia, esophagitis, bodily pain or general health, but with better quality of life. This data highlighted also a technical detail described in 1987 [12], which consists of the verticalization of the esophageal axis before the Heller-Dor procedure. Given these data, the LHD procedure has become a standard in the management of end-stage achalasia thanks to minimal invasiveness, and good functional results. It is noteworthy to underline that the Brazilian experience with the Chagas disease did not show limitations of the Heller myotomy in a dilated esophagus even more than 10 cm. Furthermore, it is not considered an end-stage achalasia and in consequence is not considered a contraindication of Heller myotomy and even shows good results [13]. However, we should underline that the subject is still debated especially in aperistaltic esophagus where Heller myotomy showed in different studies poor results. In these cases, esophagectomy is preferred. The latter is also discussed after the failure of a previous myotomy or after the failure of endoscopic attempts, in tortuous esophagus where the shape of the esophagus could interfere with the emptying even after a Heller myotomy [[14], [15], [16]]. We should also emphasize the importance of high-resolution manometry as it could predict the outcomes of such surgery based on the Chicago classification [17]. To our knowledge, our patient is among the few cases which challenge the conservative option in stage IV achalasia. This idea was supported by a retrospective study [6], which showed that ¾ of patients operated on for radiological stage IV achalasia, experienced significant relief in symptoms after LHD.

## 4 Conclusion

LHD could be considered as an alternative in the management of end-stage achalasia. It is less mutilating than the standard approach with excellent follow-up.

The following are the supplementary data related to this article.

Supplementary video Video of the surgical procedure. Supplementary video

## Patient consent

Written informed consent was obtained from the patient to publish this case report and accompanying images. On request, a copy of the written consent is available for review by the Editor-in-Chief of this journal.

## Funding

No funding.

## Ethical approval

Ethical approval is exempt/waived at our institution.

## Research registration number

Not applicable.

## Declaration of competing interest

The authors declare no competing interest.
